# Diabetes Risk Factors, Diabetes Risk Algorithms, and the Prediction of Future Frailty: The Whitehall II Prospective Cohort Study

**DOI:** 10.1016/j.jamda.2013.08.016

**Published:** 2013-11

**Authors:** Kim Bouillon, Mika Kivimäki, Mark Hamer, Martin J. Shipley, Tasnime N. Akbaraly, Adam Tabak, Archana Singh-Manoux, G. David Batty

**Affiliations:** a Department of Epidemiology and Public Health, University College London, London, UK; b Finnish Institute of Occupational Health, Helsinki, Finland; c INSERM U1061, Montpellier, France; d University Montpellier I, Montpellier, France; e Semmelweis University Faculty of Medicine, 1st Department of Medicine, Budapest, Hungary; f INSERM U1018, Centre for Research in Epidemiology and Population Health, Villejuif, France; g Hôpital Sainte Périne, Centre de Gérontologie, AP-HP, Paris, France; h Centre for Cognitive Ageing and Cognitive Epidemiology, University of Edinburgh, Edinburgh, UK

**Keywords:** Aging, frailty, diabetes risk scores, diabetes risk factors

## Abstract

**Objective:**

To examine whether established diabetes risk factors and diabetes risk algorithms are associated with future frailty.

**Design:**

Prospective cohort study. Risk algorithms at baseline (1997–1999) were the Framingham Offspring, Cambridge, and Finnish diabetes risk scores.

**Setting:**

Civil service departments in London, United Kingdom.

**Participants:**

There were 2707 participants (72% men) aged 45 to 69 years at baseline assessment and free of diabetes.

**Measurements:**

Risk factors (age, sex, family history of diabetes, body mass index, waist circumference, systolic and diastolic blood pressure, antihypertensive and corticosteroid treatments, history of high blood glucose, smoking status, physical activity, consumption of fruits and vegetables, fasting glucose, HDL-cholesterol, and triglycerides) were used to construct the risk algorithms. Frailty, assessed during a resurvey in 2007–2009, was denoted by the presence of 3 or more of the following indicators: self-reported exhaustion, low physical activity, slow walking speed, low grip strength, and weight loss; “prefrailty” was defined as having 2 or fewer of these indicators.

**Results:**

After a mean follow-up of 10.5 years, 2.8% of the sample was classified as frail and 37.5% as prefrail. Increased age, being female, stopping smoking, low physical activity, and not having a daily consumption of fruits and vegetables were each associated with frailty or prefrailty. The Cambridge and Finnish diabetes risk scores were associated with frailty/prefrailty with odds ratios per 1 SD increase (disadvantage) in score of 1.18 (95% confidence interval: 1.09–1.27) and 1.27 (1.17–1.37), respectively.

**Conclusion:**

Selected diabetes risk factors and risk scores are associated with subsequent frailty. Risk scores may have utility for frailty prediction in clinical practice.

Aging is associated with multisystem decline, which can lead to frailty, a clinically recognized geriatric syndrome characterized by declines in functioning across an array of physiologic systems.^1^ Frailty itself has a series of negative consequences, including a future risk of disability,^2^ institutionalization,^3^ fracture,^4^ hospitalization,^5^ and mortality.^4^, ^6^ Identification of modifiable risk factors for frailty^7^ is clearly important in the prevention of the syndrome.

One such modifiable predictor of frailty may be diabetes^8^ and its risk factors. Diabetes risk factors that have recently been shown to be related to an elevated risk of frailty include adiposity,^9^ low high-density lipoprotein (HDL)-cholesterol level,^10^ high blood pressure,^11^ and cigarette smoking.^12^

However, this evidence base is modest; studies are typically small in scale and cross-sectional in design, and the influence, if any, of other diabetes risk factors (history of high blood glucose, physical activity, consumption of fruit and vegetables, fasting glucose, and triglycerides) on future frailty is unknown. Additionally, in the clinical setting, predictive risk algorithms that are in frequent use for the purposes of predicting diabetes and that comprise these risk factors offer value in estimating the likelihood of future disease and therefore provide clinical guidance in prevention and treatment.

In the present analyses, we examined the longitudinal association between a comprehensive range of individual diabetes risk factors, validated diabetes risk algorithms (Framingham Offspring,^13^ Cambridge,^14^ and Finnish^15^), and future frailty. If a strong association between the diabetes risk scores and frailty is confirmed, these scores would present a convenient way to identify individuals at an increased risk of frailty later in life and in need of early preventive measures.

## Methods

### Study Population

Described in detail elsewhere,^16^ data were drawn from the Whitehall II study, an ongoing longitudinal study of 10,308 (67% men) London-based British civil servants aged 35 to 55 years at study induction.^17^ The first screening (phase 1) took place from 1985 to 1988, involving a clinical examination and self-administered questionnaire. Subsequent phases of data collection have alternated between postal questionnaire alone (phases 2 [1988–1990], 4 [1995–1996], 6 [2001], 8 [2006], and 10 [2011]), and postal questionnaire accompanied by a clinical examination approximately every 5 to 6 years (phases 3 [1991–1993], 5 [1997–1999], 7 [2002–2004], and 9 [2007–2009]).

We used diabetes risk factors measured at phase 5, the “baseline” for the purposes of our analyses. Frailty was assessed approximately 10 years later, at phase 9, when its components were measured for the first time. Diabetes status was assessed at phases 5, 7, and 9. Prevalent diabetes cases at phase 5 were excluded from the population. Ethical approval for the Whitehall II study was obtained from the University College London Medical School Committee on the ethics of human research (London, UK).

### Diabetes Risk Factors (1997–1999)

Lifestyle indices, anthropometric, and cardiometabolic risk factors of diabetes were considered. Smoking habit (non, former, and current), physical activity (<4 h/wk, ≥4 h/wk), and daily consumption of fruits and vegetables (yes, no) were ascertained by self-reported questionnaire.

Anthropometric measures included body mass index (BMI) (calculated by dividing weight, in kilograms, by height, in meters, squared and categorized using established classifications^18^), and waist circumference taken to be the smallest girth at/or below the costal margin. The latter was categorized as small (<94 cm in men and 80 cm in women), intermediate (94 to <102 cm in men and 80 to <88 cm in women), and high (≥102 cm in men and 88 cm in women).^19^ Cardiometabolic measures included use of antihypertensive or corticosteroid medication, measures of systolic and diastolic blood pressure, fasting and a 2-hour postload glucose, serum total and HDL-cholesterol, and serum triglycerides. Blood samples were collected following either an 8-hour overnight fast or at least a 4-hour fast after a light, fat-free breakfast. Genetic risk was proxied by having a parent or sibling with a history of diabetes.

Based on measures ascertained at the phase 5 examination, we calculated the following diabetes risk algorithms: the Framingham Offspring,^13^ the Cambridge,^14^ and the Finnish^15^ diabetes risk scores. Supplementary Table 1 summarizes the components of these models.

### The Fried Frailty Measure (2007–2009)

Comprising 5 individual components, frailty was ascertained using the Fried frailty scale in 2007 to 2009.^20^

• *Exhaustion*: defined using 2 items drawn from the Center for Epidemiology Studies-Depression (CES-D) scale^21^: “I felt that everything I did was an effort in the last week” and “I could not get going in the last week.” If participants answered “occasionally or moderate amount of the time (3–4 days)” or “most or all of the time (5–7 days)” to either of these items, they were categorized as being exhausted.
• *Physical activity*: based on a modified version of the Minnesota leisure-time physical activity questionnaire^22^ that includes 20 items on the frequency and duration of participation in different activities (eg, running, cycling, other sports, housework, and gardening activities). Total hours per week were calculated for each activity and a metabolic equivalent (MET) value was assigned to each based on a compendium of values.^23^ Energy expenditure (kcal/wk) was then computed for each participant. Low levels of physical activity were denoted by an expenditure of less than 383 kcal/wk in men and <270 in women.
• *Walking speed*: based on usual walking speed over a distance of 8 feet (2.4 meters). With established thresholds to denote risk being based on results for a 15-foot (4.6 meters) walking test, following downward calibration, participants were categorized as having slow walking speed when time to walk 8 feet for men with height ≤173 cm was ≥3.73 seconds or ≥3.20 seconds with height >173 cm. For women, slow walking time was ≥3.73 seconds with height ≤159 cm or ≥3.20 seconds with height >159 cm.
• *Grip strength*: measured using the Smedley handgrip dynamometer (Scandidact, Odder, Denmark). Thresholds are stratified by gender and BMI. For men, low grip strength was denoted as ≤29 kg (BMI ≤24 kg/m^2^), ≤30 (BMI 24.1–28.0), and ≤32 (BMI >28.0). For women, low grip strength was ≤17 kg (BMI ≤23 kg/m^2^), ≤17.3 (BMI 23.1–26.0), ≤18 (BMI 26.1–29.0), and ≤21 (BMI >29.0).
• *Weight loss*: In accordance with that in the Women's Health Aging Study-I,^24^ we used data from 2 assessments (2002–2004 and 2007–2009) to identify weight loss of greater than 10% in the intervening 5-year period.

A total frailty score was calculated by allocating a value of 1 to each of the above criteria if present (range: 0 to 5). Participants were classified as “frail” if they were positive for at least 3 of 5 of the frailty components; as “prefrail” if they had 1 to 2; and as “nonfrail” if they had none of these components.^20^

### Diabetes

To evaluate the performances of the diabetes risk scores in the prediction of future frailty, we used diabetes as a reference outcome. Type 2 diabetes was defined as fasting glucose ≥7.0 mmol/L or a 2-hour postload glucose ≥11.1 mmol/L, and/or as physician-diagnosed diabetes, and/or use of diabetes medication for those with diagnosed diabetes.^25^ To identify only incident (new) cases of diabetes, people with diabetes at the 1997–1999 screening (n = 450) were removed from the analyses.

### Statistical Analyses

Each diabetes risk factor was described according to frailty status (frail/prefrail and nonfrail) at the 10-year follow-up and compared using chi-square tests for the categorical factors and the Wilcoxon signed-rank test for the continuous factor (age only).

We then used binary logistic regression analyses to examine the associations between individual risk factors for diabetes and subsequent frailty. In these analyses, frailty status was dichotomized (frail/prefrail versus nonfrail) owing to the low number of frail participants. To test the independence of these associations, we fitted fully adjusted models using all the risk factors (age, sex, family history of diabetes, BMI, waist circumference, systolic/diastolic blood pressure, antihypertensive and corticoid treatments, smoking status, physical activity, daily consumption of fruits and vegetables, fasting glucose, HDL-cholesterol, and triglycerides). Men and women were combined in the analyses; however, as sex modified the relation of the standardized risk score with frailty for the Cambridge score (*P* values for sex interaction = .03), we also reported results stratified by sex for this score only.

Logistic regression models were also used to examine the association of diabetes risk scores with frailty. These were estimated calculating the standardized odds ratio (OR) of being frail/prefrail per 1-SD increase (higher score greater diabetes risk) in the risk scores over the 10-year follow-up. To compare the magnitude of the associations among the 3 risk scores with future frailty, we calculated a 95% confidence interval (CI) around the difference between the standardized ORs using a bias-corrected and accelerated (BCa) bootstrap method with 2000 resamplings.^26^ To place these effect estimates into context, we also related diabetes risk scores with incident diabetes.

To examine the robustness of the association between frailty/prefrailty and the diabetes risk scores, we conducted several sensitivity analyses: in a study sample excluding incident diabetes cases (sensitivity analysis 1) and in a study sample including prevalent diabetes cases (sensitivity analysis 2). As the variable assessing physical activity is included in both the Finnish score and the Fried's frailty scale, one may expect to observe a strong relationship between this score and frailty. To study the use of the diabetes scores in the prediction of frailty independent of physical activity, we conducted a further sensitivity analysis (3) using the Fried's scale without the physical activity component. In addition, we also imputed data for missing frailty status and individual diabetes risk factors included in the 3 studied diabetes risk scores for those participants who responded to both the questionnaire and attended the screening examination at baseline (n = 6510) using the method of multiple imputation by chained equations.^27^ We imputed missing values 200 times using an SAS-callable software application, IVEware^28^ (University of Michigan, Ann Arbor, MI; sensitivity analysis 4).

To evaluate the predictive power for each risk score and to estimate its clinical validity, we calculated the area under the receiver operating characteristic (ROC) curve (AUC).^29^ To explore the extent to which the relationship between the risk scores and frailty was driven by specific diabetes risk factors included in the scores, analyses on the risk scores–frailty associations were adjusted successively for the individual risk factors one at a time. All analyses were performed with SAS software, version 9.1 (SAS Institute, Inc, Cary, NC).

This study was approved by the University College London ethics committee, and participants provided written informed consent.

## Results

**Fig. 1:**
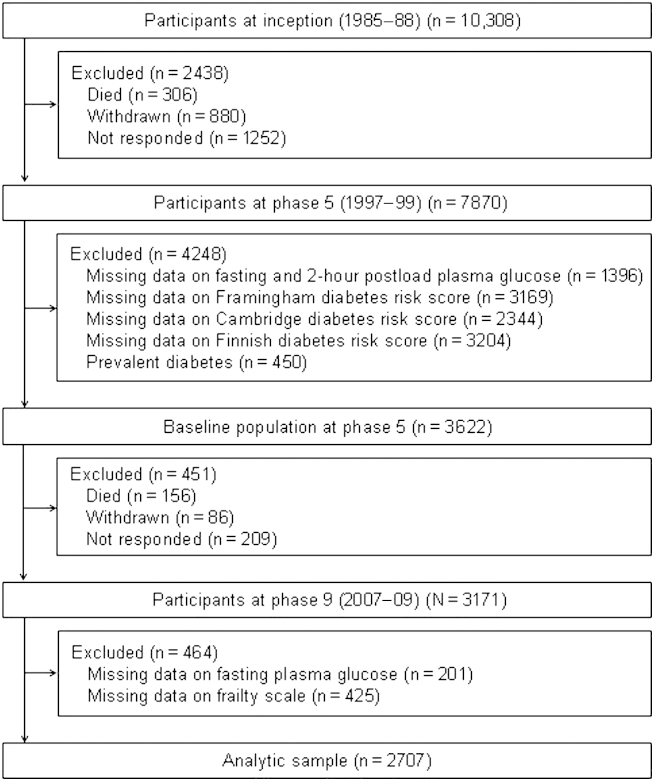
Flow of study members featured in the present analyses through the Whitehall II data collection phases.

A total of 2707 participants (755 women) aged 45 to 69 years at phase 5 constituted the analytic sample; Figure 1 shows the sample derivation. In comparison with the 5292 study members alive at phase 9 but excluded (owing to nonparticipation at phases 5 and 9 or missing data on the diabetes risk scores, plasma glucose, or the frailty scale), those included in the analytic sample were 0.3 years younger (*P* = .005), less likely to be female (27.9% versus 32.7%, *P* < .0001) and from the lower socioeconomic group (13.0% versus 22.7%, *P* < .0001).

Of the 2707 participants, 2.8% were classified as frail, 37.5% prefrail, and 59.7% nonfrail. Baseline characteristics of participants as a function of frailty status at the end of follow-up (on average 10.5 years, SD = 0.5) are detailed in Table 1. In comparison with nonfrail participants, frail/prefrail participants were more likely to be older and female; have higher BMI, waist circumference, and blood pressure; be a current smoker; and less likely to be physically active and consume fruits and vegetables on a daily basis. Frail participants were also more likely to have experienced diabetes during the follow-up relative to their nonfrail counterparts (11.2% versus 7.4%, *P* = .0006).

**Table 1:** Baseline Characteristics and Incident Diabetes in Study Participants (n = 2707)

	All	Frailty Status at Follow-up		*P* Value∗
		Not Frail	Prefrail/Frail	
Numbers	2707	1616	1091	
Age, y (SD)	55.0 (5.9)	54.6 (5.6)	55.6 (6.2)	.0005
Sex, n (%)				
Male	1952 (72.1)	1228 (76.0)	724 (66.4)	<.0001
Female	755 (27.9)	388 (24.0)	367 (33.6)	
Parental or siblings history of diabetes, n (%)				
No	2419 (89.4)	1443 (89.3)	976 (89.5)	.89
Yes	288 (10.6)	173 (10.7)	115 (10.5)	
Body mass index, kg/m^2^				.002
<25	1199 (44.3)	730 (45.2)	469 (43.0)	
25–30	1145 (42.3)	700 (43.3)	445 (40.8)	
≥30	363 (13.4)	186 (11.5)	177 (16.2)	
Waist circumference, cm				<.0001
Men: <94/women: <80	1414 (52.2)	879 (54.4)	535 (49.0)	
Men: 94–102/women: 80–88	719 (26.6)	442 (27.4)	277 (25.4)	
Men: ≥102/women: ≥88	574 (21.2)	295 (18.2)	279 (25.6)	
Blood pressure ≥130/85 mm Hg or hypertension therapy, n (%)				
No	1629 (60.2)	1005 (62.2)	624 (57.2)	.009
Yes	1078 (39.8)	611 (37.8)	467 (42.8)	
Corticosteroid treatment, n (%)				
No	2608 (96.3)	1562 (96.7)	1046 (95.9)	.29
Yes	99 (3.7)	54 (3.3)	45 (4.1)	
Smoking status, n (%)				
Nonsmoker	1514 (55.9)	891 (55.1)	623 (57.1)	.002
Ex-smoker	967 (35.7)	610 (37.8)	357 (32.7)	
Current smoker	226 (13.0)	115 (7.1)	111 (10.2)	
Low physical activity < 4 h/wk, n (%)				
No	968 (35.8)	711 (44.0)	257 (23.6)	<.0001
Yes	1739 (64.2)	905 (56.0)	834 (76.4)	
Daily consumption of fruits and vegetables, n (%)				
No	709 (26.2)	373 (23.1)	336 (30.8)	<.0001
Yes	1998 (73.8)	1243 (76.9)	755 (69.2)	
Fasting glucose level 100–126 mg/dL, n (%)				
No	2292 (84.7)	1370 (84.8)	922 (84.5)	.85
Yes	415 (15.3)	246 (15.2)	169 (15.5)	
High-density lipoprotein cholesterol, mg/dL				.06
Men: <40/women: <50	421 (15.5)	234 (14.5)	187 (17.1)	
Men: ≥40/women: ≥50	2286 (84.5)	1382 (85.5)	904 (82.9)	
Triglycerides level ≥100 mg/dL				.45
No	2109 (77.9)	1267 (78.4)	842 (77.2)	
Yes	598 (22.1)	349 (21.6)	249 (22.8)	
Incident diabetes at follow-up, n (%)				
No	2466 (91.1)	1497 (92.6)	969 (88.8)	.0006
Yes	241 (8.9)	119 (7.4)	122 (11.2)	

∗ *P* for heterogeneity based on chi-square test or Wilcoxon signed-rank test.

Supplementary Table 2 shows that older age, being a woman, physical inactivity, and no daily consumption of fruits and vegetables were independently associated with an increased risk of future frailty/prefrailty, whereas ex-smokers experienced a decreased risk.

Table 2 shows results of the association between baseline diabetes risk scores and frailty/prefrailty and incident diabetes. A 1-SD increase (disadvantage) in the Framingham and Finnish scores was associated with a 4% increase in the probability of developing diabetes. For the Cambridge score, it represented 18%. Both Cambridge and Finnish risk scores were associated with future frailty/prefrailty with OR per 1-SD increment in the score 1.18 (95% CI 1.09–1.27) and 1.27 (95% CI 1.17–1.37), respectively. The Framingham Offspring score was not associated with future frailty/prefrailty, OR = 1.05 (95% CI 0.98–1.14).

**Table 2:** Comparison of Performances of Diabetes Risk Scores∗ in the Prediction of Future Frailty and Diabetes

	Difference (Δ) in OR† (95% CI)‡ for Frailty	
	Framingham Risk Score OR = 1.05 (0.98–1.14)	Cambridge Risk Score OR = 1.18 (1.09–1.27)
Framingham risk score OR = 1.05 (0.98–1.14)	—	—
Cambridge risk score OR = 1.18 (1.09–1.27)	Δ = 0.12 (0.02–0.22)	—
Finnish risk score OR = 1.27 (1.17–1.37)	Δ = 0.22 (0.11–0.33)	Δ = 0.09 (0.02–0.17)

	Difference (Δ) in OR (95% CI)∗ for Diabetes	
	Framingham Risk Score OR = 1.72 (1.56–1.90)	Cambridge Risk Score OR = 1.69 (1.52–1.88)
Framingham risk score OR = 1.72 (1.56–1.90)	—	—
Cambridge risk score OR = 1.69 (1.52–1.88)	Δ = −0.03 (−0.28–0.21)	—
Finnish risk score OR = 1.52 (1.38–1.68)	Δ = −0.20 (−0.46–0.01)	Δ = −0.17 (−0.32 to −0.05)

CI, confidence interval; OR, odds ratio; —, not applicable.

∗ A 1-SD increase (disadvantage) in the Framingham and Finnish scores was associated with a 4% increase in the probability of developing diabetes. For the Cambridge score, it represented 18%.

† ORs are per 1-SD increment in score.

‡ Bias-corrected and accelerated bootstrap (BCa) 95% CI.

The Finnish risk score had a significantly stronger association with frailty/prefrailty than the other 2 scores, whereas the Cambridge score also showed a stronger association than the Framingham score (Table 2).

As anticipated, all risk scores were statistically associated with incident diabetes in this population, although the Finnish score had a weaker association than the other 2 scores (Table 2). The associations between the diabetes scores and frailty/prefrailty changed slightly after exclusion of incident diabetes cases over the follow-up, inclusion of prevalent diabetes, modification of the Fried's scale (original scale without physical activity component), and multiple imputations, but the ranking of their associations with frailty/prefrailty was maintained (Supplementary Table 3).

Supplementary Table 4 presents results of analyses in which the 3 diabetes scores as a whole were adjusted for each of their risk factors. For the Cambridge and Finnish scores, the association with frailty/prefrailty remained statistically significant after successive adjustments for risk factors, suggesting that this association was not driven by any one specific risk factor.

Table 3 shows the AUC for each diabetes score in the prediction of frailty/prefrailty. The Finnish score had the highest AUC compared with the other scores (0.58 versus 0.53 and 0.54 for the Framingham and Cambridge scores, respectively). In the prediction of diabetes, the Framingham score had the highest AUC (0.76 versus 0.68 and 0.70 for the Finnish and Cambridge scores, respectively).

**Table 3:** Comparisons of the AUCs and Their 95% CIs in the Prediction of Frailty and Diabetes

	Frail and Prefrail		Diabetes	
	AUC (95% CI)	Δ (95% CI)∗	AUC (95% CI)	Δ (95% CI)∗
Framingham risk score	0.531 (0.509–0.553)	0.044 (0.022–0.066)	0.760 (0.727–0.792)	Ref
Cambridge risk score	0.535 (0.513–0.557)	0.040 (0.023–0.057)	0.699 (0.666–0.732)	0.061 (0.025–0.097)
Finnish risk score	0.575 (0.553–0.597)	Ref	0.684 (0.649–0.718)	0.076 (0.040–0.112)

AUC, area under the receiver operating curve; CI, confidence interval.

∗ Difference in the AUCs.

## Discussion

In this middle-aged cohort, we examined diabetes risk factors, and various diabetes risk engines, as predictors of future frailty. Our main finding was the identification of a series of new risk factors for frailty. Moreover, we showed that risk prediction using established diabetes models was modest and smaller than that apparent for the diabetes. Risk factors associated with frailty were increased age, being female, and 2 markers of unhealthy behaviors (physical activity less than 4 hours per week and no daily consumption of fruits and vegetables) and 1 marker of healthy behavior (stopping smoking).

Age is an obvious predictor of frailty/prefrailty.^30^ Greater risk of frailty/prefrailty among women is also well known.^30^ The strong relationship between physical inactivity and subsequent frailty/prefrailty is to be expected given that it is also 1 of the 5 components of Fried's frailty measurement.^20^ However, frailty/prefrailty defined with the Fried's scale without the physical activity component showed a similar level of association. This association is also plausible because inactivity is related to an accelerated loss of lean mass due to a decrease in muscle fibers leading to a low physical capability.^31^ One plausible mechanism linking fruit and vegetable consumption and frailty may be the antioxidant effect of nutrients in fruits and vegetables, such as carotenoids, vitamins (C, E), and phenolics. These antioxidants have been shown to inhibit lipid peroxidation in vitro, particularly that of low-density lipoproteins (LDL)^32^ responsible for the development of atherosclerosis,^33^ the primary cause of cardiovascular diseases, which have been shown to be related to frailty in several cross-sectional studies.^34^ Although several prospective studies demonstrated that fruit and vegetable consumption is protective against noncommunicable diseases, particularly cardiovascular diseases,^35^ the beneficial effect may not be due to isolated individual antioxidant compounds included in fruits and vegetables, as important meta-analyses of randomized controlled trials failed to show a beneficial effect of vitamins E, C, or β-carotene,^36^ rather joint effects of known or unknown antioxidants. In addition, we cannot rule out other mechanisms besides the antioxidant effect that explain such associations. Several researchers support the notion that fruit and vegetable intake is a marker of healthy lifestyle behavior rather than an etiological factor of noncommunicable diseases, as it is highly correlated with other disease risk factors.^37^ Although a few studies found that smokers are at high risk of frailty/prefrailty,^38^, ^39^ to our knowledge, no other studies have reported a beneficial effect of stopping smoking on frailty/prefrailty. This positive healthy behavior was also observed in this study when looking at cognitive function: ex-smokers had lower risk of poor cognition.^40^ Greater beneficial health effects among those who give up smoking compared with nonsmokers may be due to a greater improvement in other health behaviors.

The higher magnitude of association and prediction between the Finnish score and frailty may be due to its composition: this model included the risk factors that were more strongly associated with frailty as seen previously in this article. This association was not driven by any one specific risk factor included in this score. In particular, physical inactivity, which is also included in the operationalization of the Fried frailty measure, was not solely responsible for the stronger association. Smaller associations of the Cambridge and Framingham risk scores with frailty may be explained by the effect of sex, as the direction of the association was unexpected in the prediction of frailty. In addition, 3 strong predictors of frailty were not included. Indeed, old women are more likely to become frail than old men,^30^ whereas in the prediction of diabetes, sex has a nonsignificant effect in the Framingham score (β for men = −0.01) and women are less at risk in the Cambridge score (β for women = −0.88).

Our study has some limitations. First, we identified frailty cases using a measure operationalized by Fried and colleagues,^20^ but a recent review identified more than 20 alternative measures of frailty.^41^ Although there are no gold standard measures, the measure by Fried and colleagues^20^ is the most widely used. Second, contrary to cardiovascular diseases whose gold standard risk score is the Framingham risk score and that is routinely used in clinical and public health practice, there is no such gold standard for diabetes. Although there are numerous diabetes risk scores, they are less known and used.^42^ However, in the literature, the 3 risk scores that we used were widely validated and well known compared with other diabetes risk scores. Third, our study sample consisted of middle-aged civil servants, limiting the generalizability of our findings. However, these limitations can be compared with the main strength of our study, which resides in the use of prospectively collected data that allowed us to test an original hypothesis.

## Conclusion

In conclusion, diabetes risk scores, in particular the Finnish score, were associated with future frailty. Our findings may help in the construction of an original prediction model to identify middle-aged persons at risk of frailty.
