# Supplementary material for: Diabetes Risk Factors, Diabetes Risk Algorithms, and the Prediction of Future Frailty: The Whitehall II Prospective Cohort Study
Source: J Am Med Dir Assoc. 2013 Nov;14(11):851.e1–6. doi: 10.1016/j.jamda.2013.08.016 (PMC3820037; doi:10.1016/j.jamda.2013.08.016)
Supplement: Supplementary Tables 1–4 [file mmc1.doc]

**Supplementary Table 1**

Components Comprising the Framingham Offspring, Cambridge, and Finnish Diabetes Risk Scores

| Study | Year | Country | Age | Sex | PSHD | BMI | WC | SBP | DBP | AHT | Steroid | HHG | Smoking | PA | FV | FG | HDL | TG |
| --- | --- | --- | --- | --- | --- | --- | --- | --- | --- | --- | --- | --- | --- | --- | --- | --- | --- | --- |
| Framingham | 2007 | USA | X | X | X | X |  | X | X | X |  |  |  |  |  | X | X | X |
| Cambridge | 2000 | UK | X | X | X | X |  |  |  | X | X |  | X |  |  |  |  |  |
| Finnish | 2003 | Finland | X |  |  | X | X |  |  | X |  | X |  | X | X |  |  |  |

AHT, antihypertensive treatment; BMI, body mass index; FG, fasting glucose; FV, daily consumption of fruits and vegetables; HDL-C, High-density lipoprotein cholesterol; HHG, history of high blood glucose; PA, physical activity < 4 h/wk; PSHD, parent/sibling history of diabetes; SBP, systolic blood pressure; TC, total cholesterol; TG, triglycerides; WC, waist circumference.

**Supplementary Table 2**

Odds Ratio (95% Confidence Interval) for the Association Between Individual Components of the Diabetes Risk Scores and Frailty (n = 2707)

|  | Adjusted for Sex | Fully Adjusted* |
| --- | --- | --- |
| Age, y | 1.03 (1.01–1.04) | 1.04 (1.03–1.06) |
| Sex |  |  |
| Male | — | 1 (ref) |
| Female | — | 1.29 (1.07–1.56) |
| Parental/sibling history of diabetes |  |  |
| No | 1 (ref) | 1 (ref) |
| Yes | 0.95 (0.74–1.22) | 0.85 (0.65–1.10) |
| BMI, kg/m**^2^** |  |  |
| <25 | 1 (ref) | 1 (ref) |
| 25–30 | 1.02 (0.87–1.21) | 0.94 (0.76–1.17) |
| ≥30 | 1.42 (1.12–1.80) | 1.10 (0.76–1.58) |
| Waist circumference, cm |  |  |
| Men: <94/women: <80 | 1 (ref) | 1 (ref) |
| Men: 94–102/women: 80–88 | 1.03 (0.86–1.24) | 0.99 (0.79–1.25) |
| Men: ≥102 / women: ≥ 88 | 1.44 (1.18–1.76) | 1.25 (0.91–1.71) |
| Blood pressure (≥130/85 mm Hg) or hypertension therapy use |  |  |
| No | 1 (ref) | 1 (ref) |
| Yes | 1.25 (1.06–1.46) | 1.09 (0.92–1.29) |
| Corticoid treatment |  |  |
| No | 1 (ref) | 1 (ref) |
| Yes | 1.27 (0.85–1.91) | 1.22 (0.80–1.85) |
| Smoking status |  |  |
| Nonsmoker | 1 (ref) | 1 (ref) |
| Ex-smoker | 0.87 (0.74–1.03) | 0.82 (0.69–0.97) |
| Current smoker | 1.35 (1.02–1.79) | 1.17 (0.87–1.57) |
| Low physical activity (< 4 h/wk) |  |  |
| No | 1 (ref) | 1 (ref) |
| Yes | 2.41 (2.02–2.87) | 2.49 (2.08–2.98) |
| Daily consumption of fruits and vegetables |  |  |
| No | 1.57 (1.32–1.87) | 1.51 (1.26–1.82) |
| Yes | 1 (ref) | 1 (ref) |
| Fasting glucose, 100–126 mg/dL |  |  |
| No | 1 (ref) | 1 (ref) |
| Yes | 1.06 (0.86–1.32) | 0.99 (0.79–1.24) |
| HDL cholesterol, mg/dL |  |  |
| Men: <40/women: <50 | 1.22 (0.99–1.51) | 1.08 (0.86–1.36) |
| Men: ≥40/women: ≥50 | 1 (ref) | 1 (ref) |
| Triglycerides level ≥100 mg/dL |  |  |
| No | 1 (ref) | 1 (ref) |
| Yes | 1.15 (0.96–1.39) | 0.98 (0.80–1.21) |

BMI, body mass index; CI, confidence interval; HDL, high-density lipoprotein; OR, odds ratio.

*Model includes all predictors in addition to sex.

**Supplementary Table 3**

Sensitivity Analyses: Odds Ratio (95% Confidence Interval) per 1-SD Increment in Score Using 3 Diabetes Risk Algorithms for Future Frailty

| Diabetes Risk Scores | Main Analysis | Sensitivity Analysis 1 | Sensitivity Analysis 2 | Sensitivity Analysis 3 | Sensitivity Analysis 4 |
| --- | --- | --- | --- | --- | --- |
|  | Study Sample  (n = 2707) | Study Sample Excluding Incident Diabetes Cases (n = 2466) | Study Sample Including Prevalent Diabetes Cases (n = 2824) | Fried’s Frailty Scale Excluding Physical Activity Component (n = 2697) | Multiple Imputation (n = 6510) |
| Framingham Offspring risk score | 1.05 (0.98–1.14) | 1.07 (0.97–1.18) | 1.09 (1.01–1.17) | 1.06 (0.97–1.15) | 1.11 (1.05–1.18) |
| Cambridge risk score | 1.18 (1.09–1.27) | 1.14 (1.04–1.23) | 1.18 (1.09–1.27) | 1.23 (1.13–1.33) | 1.17 (1.10–1.25) |
| Finnish risk score | 1.27 (1.17–1.37) | 1.25 (1.14–1.36) | 1.26 (1.16–1.37) | 1.28 (1.18–1.39) | 1.24 (1.16–1.34) |

**Supplementary Table 4**

Association Between Diabetes Risk Scores Components Mutually Adjusted for Diabetes Risk Scores and Frailty

| Diabetes Risk Scores | OR (95% CI) |
| --- | --- |
| Framingham diabetes score |  |
| Unadjusted | 1.05 (0.98–1.14) |
| Adjusted for |  |
| Age | 1.05 (0.97–1.13) |
| Sex | 1.06 (0.98–1.14) |
| Parental history of diabetes | 1.05 (0.97–1.14) |
| BMI | 1.02 (0.94–1.11) |
| Blood pressure ≥ 130/85 mm Hg or receiving therapy | 1.03 (0.95–1.11) |
| HDL-cholesterol | 1.03 (0.95–1.12) |
| Triglycerides | 1.05 (0.97–1.13) |
| Fasting glucose | 1.09 (0.98–1.21) |
| Cambridge diabetes score |  |
| Unadjusted | 1.18 (1.09–1.27) |
| Adjusted for |  |
| Age | 1.14 (1.05–1.23) |
| Sex | 1.22 (1.13–1.31) |
| Parental/sibling history of diabetes | 1.19 (1.10–1.28) |
| BMI | 1.18 (1.07–1.30) |
| Antihypertensive therapy | 1.16 (1.06–1.26) |
| Corticosteroid therapy | 1.18 (1.09–1.28) |
| Smoking | 1.16 (1.07–1.25) |
| Finnish diabetes score |  |
| Unadjusted | 1.27 (1.17–1.37) |
| Adjusted for |  |
| Age | 1.25 (1.15–1.35) |
| BMI | 1.45 (1.28–1.63) |
| Waist circumference | 1.40 (1.26–1.54) |
| Antihypertensive therapy | 1.27 (1.17–1.38) |
| Physical activity <4 h/wk | 1.20 (1.11–1.30) |
| Daily consumption of fruits and vegetables | 1.26 (1.16–1.36) |

BMI, body mass index; CI, confidence interval; HDL, high-density lipoprotein; OR, odds ratio.
